# Exploring the Use of Machine Learning in Marine Biomonitoring: Assessing Nickel Exposure in *Paracentrotus lividus* Embryos

**DOI:** 10.3390/toxics14070557

**Published:** 2026-06-26

**Authors:** Lehel Dénes-Fazakas, Gaspare Drago, Andrea De Gaetano, Levente Kovács, László Szilágyi, Rosa Bonaventura

**Affiliations:** 1 Physiological Controls Research Centre, Obuda University, Bécsi út 96/b, H-1034 Budapest, Hungary; andrea.degaetano@cnr.it (A.D.G.); kovacs@uni-obuda.hu (L.K.); or szilagyi.laszlo@uni-obuda.hu (L.S.); 2 Istituto per la Ricerca e l’Innovazione Biomedica (CNR-IRIB), Consiglio Nazionale delle Ricerche, Via U. La Malfa 153, 90146 Palermo, Italy; gaspare.drago@irib.cnr.it; 3 Faculty of Technical and Human Sciences, Sapientia Hungarian University of Transylvania, Șoseaua Sighișoarei 1/C, 540485 Targu Mures, Romania

**Keywords:** convolutional neural networks, image classification, ecotoxicology, sea urchin embryos, echinoderms, pollution, data augmentation

## Abstract

The sea urchin embryo represents a well-established model organism widely applied in embryo toxicity assays, ecotoxicological investigations, and biomonitoring studies. These analyses are primarily based on the morphological evaluation of embryos, which is conventionally carried out by experts through light microscopy. However, this approach is both time-intensive and inherently subjective, as the outcomes strongly depend on the evaluator’s expertise and experience. With the increasing adoption of machine learning techniques in image classification tasks, this study investigates the applicability of machine-learning-based approaches for the classification of sea urchin embryo images. The dataset used in this work originates from a previous study examining the effects of nickel exposure on *Paracentrotus lividus* embryos, with concentrations ranging from 0.01 to 3.0 mM. Given the limited size of the available dataset, data augmentation techniques were applied to artificially expand the number of training samples. Subsequently, a convolutional neural network classification model was developed using both original and augmented images, and its performance was assessed using multiple evaluation metrics. The proposed model demonstrated strong performance, achieving a maximum F1 score of 0.976 and an accuracy of 0.988. These results indicate that machine-learning-based approaches can effectively support the classification of sea urchin embryo images even in data-constrained scenarios. Overall, this work contributes to the development of automated and objective methods for morphological assessment, with the potential to enhance both the reliability and efficiency of traditional evaluation procedures.

## 1. Introduction

Anthropogenic activities have undeniable side effects on the environment, with coastal waters being particularly highly impacted by a wide range of human activities [1]. Nowadays, it is widely acknowledged that evaluating the biological effects of contaminants requires the use of bioassays in addition to chemical analyses. Ecotoxicological batteries using invertebrates from different taxa provide a realistic biological response to evaluate the impact of chemical pollutants in the environment [2]. In many countries, environmental legislation recognizes and requires the use of these tools. At the European level, monitoring programs of chemical contaminants are recommended to integrate biological measurements of their effects on marine organisms [3].

Among these organisms, the sea urchin embryo is an excellent model for ecotoxicological studies, where it is used to evaluate the toxicity of many pollutants by monitoring morphological perturbations and molecular defence systems [4,5,6]. A variety of morphological abnormalities have been identified following exposure to different contaminants, including heavy metals [7,8,9], organic compounds [10,11], micro and nanoplastics [12,13,14], and environmental stressors such as climate change [15].

Morphological analysis is performed by expert biologists using microscopy, where embryos are categorized as normal or abnormal. Detailed classification includes the evaluation of skeletal organization, pigment cells, gut formation, and other structural features [7,16]. These analyses generate large numbers of high-resolution images, which are subsequently analyzed manually and typically allow the measurement of only a few traits at a time.

Deep learning methods have recently been applied in many domains due to their ability to process image data efficiently. Applications include medical image analysis [17,18,19], ocean data processing [20], and applications in ecology and evolutionary biology [21]. This latest review provides an in-depth overview of computer vision, a branch of artificial intelligence (AI) used to automatically extract and process information from digital images presenting both machine learning and traditional analytical techniques. For example, machine-learning-based computer vision has been used to accurately automate landmark data collection from morphometric datasets of 3D structures such as fly wings, sea bass, and bryozoan colonies [22]. A deep learning model named KimmelNet was trained using 2D bright-field images to quantify developmental delays between two populations of zebrafish embryos [23]. In another recent study, bright-field images of sea urchin larvae at 48 h post-fertilization (hpf), including embryos both unexposed (control) and exposed to four compounds at five different concentrations, were used to build the training and test datasets for the classification model [24]. Morphological shape parameters of each larva were extracted to parameterize the images and predict malformation levels. After validation of both the classification model and the size-increase measurement, the authors developed an application called SETApp to automate the sea urchin embryo test pipeline [24].

In this context, the aim of this study is to evaluate the use of a machine-learning-based method to distinguish among the different morphological types of sea urchin embryos induced by nickel exposure. These morphological abnormalities are widely used as indicators of developmental toxicity because they reflect disruptions in key biological processes, including skeletogenesis, pigment cell differentiation, and embryonic pattern formation.

## 2. Materials and Methods

### 2.1. Images of Sea Urchin Embryos Exposed to Nickel

In this study, we used images of sea urchin embryos exposed to nickel that were obtained in a previous work [25]. In that study, NiCl_2_ was added to embryos 30 min after fertilization at final concentrations of 0.01, 0.03, 0.04, 0.08, 0.5, 0.8, 1.0 and 3.0 mM. The exposure to different nickel concentrations resulted in several alterations in embryo development, including inhibition of skeleton growth and changes in the presence of pigment cells. These effects were evaluated at the endpoint of 48 h post-fertilization.

Based on these observations, embryos were classified into different morphological categories as previously described [25]. These categories include normal embryos as well as several abnormal morphotypes that differed in skeletal organization and cellular features. The classification criteria took into account the presence or absence of skeletal elements, abnormalities in skeleton structure, and the presence or absence of pigment cells.

**Figure 1:**
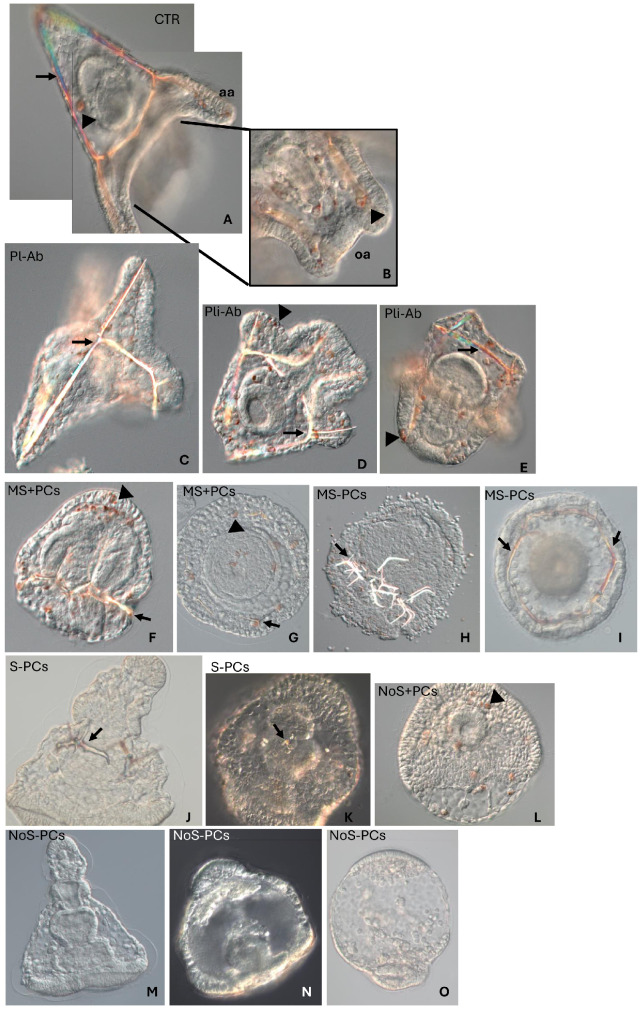
Morphotypes of Ni-treated *P. lividus* embryos at 48 hpf. Untreated embryo: (**A**) aboral view of a control embryo (CTR) at the pluteus stage, shown as a composite of two images of the same embryo; aboral arms (aa). (**B**) Detail of the oral arms (oa), which are out of focus in panel A. Morphotypes of embryos treated with different NiCl_2_ concentrations (see also Table 1): (**C**–**O**). Arrowheads indicate pigment cells (PCs); arrows indicate the embryonic skeleton. Panels K and N show embryos observed using differential interference contrast (DIC) microscopy to better visualize the skeleton.

The image dataset consists of high-resolution microscopy recordings collected during the experiments. Most of the data were stored as Tagged Image File Format files, while some images were saved in JPEG format. In addition, short video recordings were available in MPEG Transport Stream format, from which individual frames were extracted and used in the analysis. All images used in this study originate from the same experimental setup described in the reference work [25]. The images of embryos exposed to Ni at concentrations of 0.02, 0.6, 0.7, and 0.9 mM are unpublished data kindly provided by Rosa Bonaventura. The morphological categories investigated in this study, together with their corresponding biological characteristics, are summarized in Table 1 and illustrated in Figure 1. All images were visually inspected prior to analysis. No severely blurred or corrupted images were identified that required exclusion from the dataset. Furthermore, all images were acquired using the same experimental setup and were subsequently resized to a uniform resolution of 64 × 64 pixels before training.

### 2.2. Data Generator

The aim of this study is to develop a machine-learning-based approach that can distinguish between different morphological types of sea urchin embryos associated with various levels of nickel exposure. Based on the experimental conditions and the observed morphologies reported in the reference study [25], a total of 33 classes were defined. These classes represent combinations of nickel concentration and morphological category, including a control group with no exposure.

The available dataset contains 5557 images. Most of these were extracted from video recordings, while a smaller portion consists of individual microscopy images. The number of samples is relatively limited for training a convolutional neural network; therefore, data augmentation was applied to increase the size of the dataset.

Image augmentation was performed using the ImageDataGenerator class from the Keras library. This process generates modified versions of the original images through a series of random transformations. As a result, the dataset was expanded to 167,656 images, with each class represented by approximately 5000 samples. The model was trained using both original and augmented images, while evaluation was carried out exclusively on the original dataset.

All images were resized to 64 × 64 pixels before training. This resolution was selected to reduce computational cost while preserving the essential visual features required for classification [26].

The augmentation process included several types of transformations. Images were randomly rotated up to 180 degrees. Horizontal and vertical shifts were applied with a maximum displacement corresponding to 30 percent of the image size. Brightness was adjusted within a range of 0.5 to 1.5 to simulate variations in illumination. In addition, random zoom operations were applied within a range of 20 percent. Horizontal and vertical flipping were also enabled. During augmentation, each image could undergo any combination of these transformations, resulting in a diverse set of training samples. The full list of the 33 classes used for classification is presented in Table 2. The augmentation methods were selected to preserve biologically relevant morphological structures. Transformations that could artificially modify embryo morphology, such as elastic deformations or aggressive geometric distortions, were intentionally excluded. The classification task was formulated as a morphology exposure category recognition problem. Each class represents an experimentally observed combination of nickel exposure condition and embryo morphology. Thus, the objective of the model was not to estimate nickel concentration independently from image data, but to automatically assign each embryo image to one of the predefined categories used in the experimental dataset. These categories collectively include all observed morphological phenotypes, namely, CTR, PlAb, PliAb, MS + PCs, MS-PCs, S-PCs, NoS + PCs, and NoS-PCs. The augmentation strategy was designed to reduce class imbalance and improve model generalization. Each class was augmented independently until approximately 5000 samples were available per category. The selected transformations were restricted to operations that preserve the underlying biological morphology of the embryos. More aggressive augmentations, including elastic deformations, color manipulations, shearing, and other geometry-altering transformations available in the TensorFlow ImageDataGenerator framework, were intentionally excluded to maintain biological plausibility.

### 2.3. Neural Network

**Figure 2:**
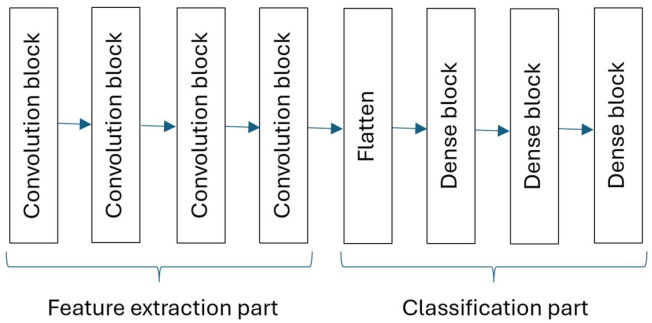
The structure of the neural network model involved in this study.

The classification model used in this study is based on a convolutional neural network architecture inspired by the Visual Geometry Group network [27]. The network is composed of two main parts. The first part is responsible for extracting relevant features from the input images, while the second part performs the classification based on these features Figure 2.

The feature extraction stage consists of a sequence of convolutional blocks. Each block includes a two-dimensional convolution with a kernel size of 3 × 3, followed by a max pooling operation and batch normalization. The activation function used in the convolutional layers is the exponential linear unit [28]. Padding was applied in such a way that the spatial dimensions are preserved after convolution, while the pooling operation reduces the size of the feature maps.

Four convolutional blocks were used in total. As the data passes through these blocks, the spatial resolution of the input image decreases, while the number of feature maps increases. Starting from an input size of 64 × 64 pixels, the representation is gradually reduced to 4 × 4 feature maps. The output of the final convolutional block is then transformed into a one-dimensional feature vector using a flattening layer.

**Figure 3:**
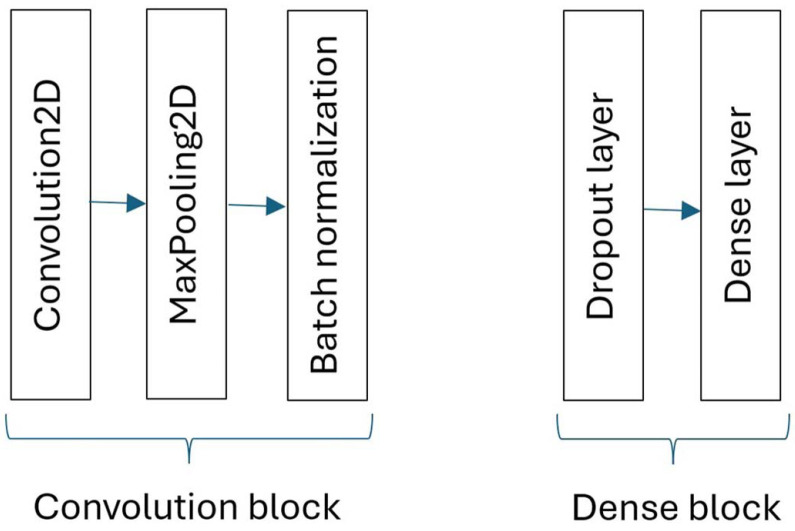
The structures of different blocks in the neural network.

The classification stage is implemented using the fully connected architecture shown in Figure 3. Two dense layers are applied before the final output layer. The number of neurons in these layers was treated as a tunable parameter and tested with several values. Dropout with a rate of 0.2 was applied after each dense layer in order to reduce overfitting. The activation function used in these layers is the rectified linear unit.

The final layer contains 33 neurons, corresponding to the number of classes in the dataset. A softmax activation function is used to produce class probabilities at the output.

Two main parameters were varied during the experiments. The first parameter is the number of filters in the first convolutional block, which was set to either 32 or 64. In each subsequent block, the number of filters was doubled. The second parameter is the number of neurons in the dense layers, which was tested with values of 32, 64, 128 and 256.

The network was trained using the Adam optimizer [29]. The loss function was sparse categorical cross-entropy. Training was performed for 100 epochs. The best performing model checkpoint was selected during training, while the final performance metrics were calculated on original non-augmented test images.

### 2.4. Evaluation Metrics

The performance of the classification model was assessed using several commonly used metrics in machine learning. These metrics provide complementary information about the behaviour of the model and help to better understand its strengths and limitations.

Precision and recall were calculated for each class. Precision reflects how many of the predicted samples belonging to a given class are correctly classified, while recall indicates how many of the actual samples of that class are successfully identified by the model. Since the dataset contains multiple classes with different numbers of samples, these metrics are particularly important for evaluating class-level performance.

The F1 score was also computed as the harmonic mean of precision and recall. This metric provides a balanced measure of performance and is especially useful when dealing with imbalanced datasets.

In addition to these measures, the area under the receiver operating characteristic curve was calculated. This metric reflects the ability of the model to distinguish between classes across different decision thresholds.

Finally, overall accuracy was used to measure the proportion of correctly classified samples among all predictions. While accuracy provides a general indication of performance, it may be influenced by class imbalance; therefore, it was interpreted together with the other metrics.

The reported results represent the average values of the metrics across all classes.

### 2.5. Development Environments

In this study, we use Python 3.8 language in a hosted cloud environment. We utilize the following platforms and libraries: Tensorflow 2.0.0, Scikit-learn Numpy, and Pandas. The implementation is achieved by using Jupyter Notebook 7.3.3 development user interface. The already mentioned hosted cloud environment is Google’s CoLaboratory (shortly “Colab”) where we apply the host provided by Google, which is free of charge by default. The dedicated resource on the platform varies from use to use, although it is around 12.69 GB VRAM and 107.79 GB VSPACE with 4 VCPUs provided by a Python 3 Google Compute Engine server.

## 3. Results

### Discrimination of Nickel Induced Embryo Morphologies Using Machine Learning

Sea urchin embryo development is perturbed in a very specific manner by 0.5 mM Ni, which causes the production of multiple skeletal elements (spicules) instead of the two spicules synthesized under physiological conditions, determining the “radialization” of the embryo [30,31]. In addition, depending on the doses, Ni determines a variety of morphotypes, e.g., with or without supernumerary spicules and with or without pigment cells [25]. Here, we analysed the images obtained at the endpoint of 48 h post-fertilization (48 hpf). At this time, untreated embryos were normal plutei (Pl) with a well-developed skeleton, a tripartite gut and pigment cells spread within the ectodermal layer (Figure 1A,B, aboral (A) and oral (B) views). Embryos exposed to different Ni concentrations from fertilization onwards showed many types of abnormal morphologies at 48 hpf, as reported in Table 1 and shown in Figure 1. Moreover, different morphotypes can be observed at the same Ni concentration. For example, as previously described [25] embryos exposed to 0.03 mM Ni showed three morphotypes: radialized embryos with multiple spicules (MS) and some pigment cells (+PCs) (see Figure 1F,G, MS+PCs), which were the predominant morphology; bell-shaped embryos with a radial organization of the multiple spicules (MS) and no pigment cells (-PCs) (Figure 1H,I, MS-PCs); and those with an early pluteus-like shape (Pli) but with an abnormal (Ab) skeleton pattern and visible pigment cells (Figure 1D,E, Pli-Ab). A small number of embryos were normal and early pluteus (Pl/Pli) Figure 1A.

Based on these observations, embryos were grouped into 33 classes, as described in the Materials and Methods Section. These classes reflect both the concentration of nickel and the observed morphological characteristics.

The evaluation of the model was performed using a five-fold cross-validation scheme. The original set of 5557 images was divided into five subsets. In each iteration, one subset was used for testing, while the remaining subsets together with their augmented versions were used for training. Only original images were included in the testing phase.

Eight different configurations of the neural network were tested by varying the number of filters in the first convolutional layer and the number of neurons in the dense layers. For each configuration, predictions were summarized in confusion matrices, from which the evaluation metrics were calculated.

The results are presented in Table 3. Among the tested configurations, the model using 64 filters in the first convolutional block and 256 neurons in the dense layers achieved the best overall performance. This configuration reached the highest values across most evaluation metrics, including F1 score, area under the curve and overall accuracy.

A second configuration, with 32 filters and 128 neurons, also showed strong performance, although slightly lower than the best model. In contrast, the configuration with 32 filters and 256 neurons performed noticeably worse than the others, suggesting that this combination of parameters is not optimal under the given training conditions.

In general, models with a higher number of filters in the convolutional layers tended to perform better. Increasing the number of neurons in the dense layers also improved performance up to a certain point, after which no further benefit was observed. The relationship between precision and recall indicates that recall values were often higher, which can be explained by the imbalance between classes in the dataset.

The results also show a strong agreement between F1 score and the area under the curve, indicating consistent model behaviour across different evaluation measures. Although overall accuracy provides a useful summary, it does not always reflect the detailed performance of the model due to class imbalance.

Overall, the proposed approach achieved high classification performance, demonstrating that the selected architecture is suitable for distinguishing between different morphological classes of sea urchin embryos.

## 4. Discussion

The results of this study show that the proposed neural network architecture can effectively support the identification of nickel-induced morphological changes in sea urchin embryos based on microscopy images. The model achieved strong performance, indicating that the visual features associated with different developmental outcomes can be reliably captured and interpreted by a data-driven approach.

The analysis of the tested configurations suggests that the balance between the number of filters in the convolutional layers and the number of neurons in the dense layers plays an important role in model performance. The best results were obtained when the dense layers contained approximately four times as many neurons as the number of filters in the first convolutional layer. This observation provides a practical guideline for selecting suitable model parameters in similar classification tasks.

The input image size of 64 × 64 pixels proved to be sufficient for capturing the relevant morphological characteristics of the embryos. Increasing the image resolution would likely require a more complex model and higher computational cost, without necessarily leading to a significant improvement in classification performance. This highlights the importance of selecting an appropriate trade off between model complexity and efficiency.

Although macro-averaged metrics provide a balanced evaluation across all classes, future studies should include class-specific analyses and confusion matrices to better characterize the types of classification errors and the relative difficulty of individual morphology–exposure categories.

Although more recent architectures such as Transformer-based models have shown promising results in image analysis tasks [32], their application in this context would require a substantially larger dataset. The relatively limited number of available images therefore represents an important constraint of the present study. As a consequence, the generalization of the model to other datasets, imaging conditions, or sea urchin species should be investigated in future work.

Nickel is a well-known environmental contaminant that can enter marine ecosystems through both natural processes and human activities [33]. Its effects on sea urchin development have been studied for several decades [30,31]. Exposure to nickel during early development can alter the normal pattern of skeleton formation and lead to the loss of bilateral symmetry, a phenomenon often referred to as radialization. The extent of these effects depends on both the concentration of nickel and the species under investigation [25].

The classification of morphological abnormalities in sea urchin embryos has long been used as a reliable indicator of environmental stress and toxicity. These morphological changes can be interpreted as biological responses to external factors and are often considered equivalent to specific biomarkers due to their direct relationship with contaminant exposure [16,34].

Recent studies have explored the use of machine learning methods to support the analysis of embryo morphology. For example, an image-based approach has been proposed to quantify developmental changes and automate the assessment of toxicity effects [24]. In addition, machine learning has been applied to high-content imaging of genetically modified embryos, demonstrating its potential in large-scale screening applications [35].

A direct comparison with previously published sea urchin embryo analysis tools remains challenging because most existing approaches focus on morphometric measurements, toxicity assessment, or image-assisted analysis rather than multi-class image classification of morphology–exposure categories. Furthermore, publicly available benchmark datasets for this specific task are currently lacking. Future studies should investigate comparative evaluations involving both conventional machine learning methods and alternative deep learning architectures.

In this context, the present study further supports the use of machine learning as a complementary tool in ecotoxicological research. The proposed approach demonstrates that CNN-based image analysis can successfully distinguish between experimentally observed morphology–exposure categories and may contribute to the standardization and automation of image evaluation workflows. Such methods have the potential to facilitate the processing of large image datasets, which is particularly relevant for monitoring programs and environmental assessments. However, dedicated studies comparing expert assessments and machine learning predictions will be required to quantify potential improvements in efficiency, reproducibility, and observer-related variability.

The broader implications of this work are aligned with the principles of the One Health approach, which emphasizes the interconnected nature of environmental, animal and human health [36]. The integration of automated analysis tools into biomonitoring frameworks can contribute to more efficient detection of environmental risks and support informed decision-making in ecosystem management.

The morphological phenotypes considered in this study are not merely visual categories but represent biologically relevant developmental outcomes associated with nickel toxicity. For example, abnormal pluteus-like phenotypes (PlAb and PliAb) indicate perturbations in normal skeletal development, while embryos exhibiting multiple spicules (MS+PCs and MS-PCs) reflect alterations in dorsoventral patterning and radialization processes previously associated with nickel exposure. The absence of spicules (NoS+PCs and NoS-PCs) represents a more severe disruption of skeletogenesis, whereas embryos with a single spicule (S-PCs) indicate partial impairment of normal skeletal formation. Consequently, the ability of the proposed model to discriminate among these phenotypes is directly related to the identification of biologically meaningful developmental abnormalities relevant to ecotoxicological assessment.

A limitation of the present classification framework is that the model was trained on combined morphology–exposure categories rather than on morphology-only labels. From an ecotoxicological perspective, the primary endpoint of sea urchin embryo bioassays is the identification of abnormal developmental phenotypes. Therefore, future work should also investigate morphology-only classification, in which images are grouped exclusively according to embryo morphology regardless of nickel concentration. In addition, the relatively limited size of the original dataset required the use of extensive data augmentation to balance the classes and increase the number of training samples. Although the selected augmentation operations were restricted to biologically plausible image transformations, augmented images cannot fully reproduce the natural biological variability observed in independent experiments. Furthermore, the image dataset was derived from a previously published experimental study and therefore reflects only the exposure conditions investigated in that work. As a consequence, the present model has not been validated on environmentally relevant low-dose nickel exposures, where developmental alterations may be more subtle and difficult to detect. Another limitation is that the proposed framework was developed and evaluated using images of a single sea urchin species (*Paracentrotus lividus*) exposed exclusively to nickel. Therefore, the generalization of the model to other contaminants, exposure scenarios, or echinoderm species remains to be investigated. Future studies should therefore validate the proposed approach on larger independently collected datasets, including environmentally relevant exposure levels, additional contaminants, and multiple biological model systems. Nevertheless, the present formulation preserves the full experimental context of the dataset and demonstrates that the CNN can distinguish the complete set of observed morphology–exposure categories with high accuracy.

## 5. Conclusions

Environmental pollution in marine ecosystems remains a major concern that requires effective and scalable monitoring strategies [37]. In this context, the present study demonstrates that machine learning can be successfully applied to the analysis of sea urchin embryo morphology for the assessment of nickel-induced developmental effects.

The results show that a convolutional neural network can achievepromising performance even when trained on a relatively limited dataset, provided that appropriate data augmentation techniques are applied. This suggests that similar approaches may be used in other ecotoxicological studies where data availability is restricted.

One of the main advantages of the proposed method is that it reduces the reliance on manual evaluation, which is often time-consuming and dependent on expert judgement. By providing a consistent and objective analysis framework, machine-learning-based approaches can support more reliable assessment of morphological changes associated with environmental stress.

In addition to improving efficiency, such methods offer flexibility in adapting to new types of pollutants and experimental conditions. This is particularly important in the context of emerging contaminants and ongoing environmental changes, including those related to climate variability.

Overall, this study should be considered a proof-of-concept demonstrating the feasibility of CNN-based classification of nickel-associated embryo morphology exposure categories. While the obtained results indicate the potential applicability of machine learning methods for automated image analysis in ecotoxicology, the proposed framework has been evaluated only under the experimental conditions represented in the available dataset. Further validation using morphology-only labels, independent datasets, environmentally relevant concentrations, additional contaminants, and multiple biological model systems will be necessary before the method can be applied as a general marine biomonitoring tool.

A key contribution of this work is the demonstration that machine learning can successfully identify nickel-related developmental phenotypes in sea urchin embryos using a comparatively simple CNN-based framework. The obtained results indicate that reliable classification can be achieved even in data-constrained scenarios, supporting the applicability of artificial intelligence in ecotoxicological image analysis. This provides a practical foundation for the future integration of more advanced artificial intelligence approaches, including synthetic data generation using Generative Adversarial Networks (GANs), diffusion models, and multimodal generative models, as well as transformer-based image analysis architectures such as Vision Transformers and hybrid CNN Transformer models, into marine biomonitoring and ecotoxicological assessment workflows.

## Figures and Tables

**Table 1:** Morphology of Ni-exposed embryos.

Morphology	Description
CTR	Control embryos cultured without Ni until the pluteus stage.
Pl Ab	Pluteus with an abnormal skeleton pattern and visible pigment cells.
Pli Ab	Early pluteus with an abnormal skeleton pattern and visible pigment cells.
MS + PCs	Radial embryos with multiple spicules and some pigment cells.
MS − PCs	Radial embryos with multiple spicules and no pigment cells.
NoS − PCs	Embryos without spicules or pigment cells.
NoS + PCs	Embryos without spicules and with few pigment cells.
S − PCs	Embryos with one spicule in the animal part and no pigment cells.

**Table 2:** Morphology exposure categories used for CNN classification.

Number	Sample	Concentration (mM)	Morphology
0	Control group	0.00	CTR
1	1_NiCl_2_	0.01	MS + PCs
2	1_NiCl_2_	0.01	Pl Ab
3	2_NiCl_2_	0.02	MS + PCs
4	2_NiCl_2_	0.02	MS − PCs
5	2_NiCl_2_	0.02	Pl Ab
6	2_NiCl_2_	0.02	Pli Ab
7	3_NiCl_2_	0.03	MS + PCs
8	3_NiCl_2_	0.03	MS − PCs
9	3_NiCl_2_	0.03	Pli Ab
10	4_NiCl_2_	0.04	MS + PCs
11	4_NiCl_2_	0.04	MS − PCs
12	4_NiCl_2_	0.04	Pli Ab
13	5_NiCl_2_	0.08	MS + PCs
14	5_NiCl_2_	0.08	MS − PCs
15	5_NiCl_2_	0.08	NoS − PCs
16	5_NiCl_2_	0.08	S − PCs
17	6_NiCl_2_	0.20	MS + PCs
18	6_NiCl_2_	0.20	MS − PCs
19	6_NiCl_2_	0.20	NoS − PCs
20	6_NiCl_2_	0.20	S − PCs
21	7_NiCl_2_	0.50	NoS + PCs
22	7_NiCl_2_	0.50	NoS − PCs
23	7_NiCl_2_	0.50	S − PCs
24	8_NiCl_2_	0.60	NoS − PCs
25	8_NiCl_2_	0.60	S − PCs
26	9_NiCl_2_	0.70	NoS + PCs
27	9_NiCl_2_	0.70	NoS − PCs
28	10_NiCl_2_	0.80	NoS − PCs
29	10_NiCl_2_	0.80	S − PCs
30	11_NiCl_2_	0.90	NoS − PCs
31	12_NiCl_2_	1.00	NoS − PCs
32	13_NiCl_2_	3.00	NoS − PCs

**Table 3:** Classification quality benchmarks.

FFCB	DLNs	Precision	Recall	F1 Score	AUC	Accuracy
32	32	0.888750	0.913930	0.892312	0.998738	0.977146
32	64	0.820990	0.832110	0.811842	0.974262	0.946194
32	128	0.930745	0.966551	0.937127	0.999441	0.977326
32	256	0.702500	0.701028	0.675296	0.868866	0.837322
64	32	0.911132	0.944587	0.915296	0.999148	0.982365
64	64	0.824978	0.850209	0.820520	0.986852	0.910203
64	128	0.871058	0.909295	0.880000	0.996168	0.953932
64	256	0.967216	0.989707	0.976064	0.999904	0.988123

## Data Availability

The raw data supporting the conclusions of this article will be made available by the authors on request.

## Abbreviations

The following abbreviations are used in this manuscript:

AUC	Area under the curve
CNN	Convolutional neural network
CTR	Control embryos cultured without nickel exposure
DLN	Dense-layer neuron
ELU	Exponential linear unit
F1	Harmonic mean of precision and recall
FFCB	Filters in the first convolutional block
JPEG	Joint Photographic Experts Group
Keras	High-level deep learning API
MPEG	Moving Picture Experts Group
MS + PCs	Radial embryos with multiple spicules and pigment cells
MS – PCs	Radial embryos with multiple spicules and no pigment cells
Ni	Nickel
NiCl_2_	Nickel chloride
NoS + PCs	Embryos without spicules and with pigment cells
NoS – PCs	Embryos without spicules and without pigment cells
PCs	Pigment cells
Pl Ab	Pluteus-like embryos with abnormal skeleton pattern
Pli Ab	Early pluteus-like embryos with abnormal skeleton pattern
ReLU	Rectified linear unit
ROC	Receiver operating characteristic
S – PCs	Embryos with one spicule and no pigment cells
TIFF	Tagged Image File Format
